# Temporal Association between Topical Ophthalmic Corticosteroid and the Risk of Central Serous Chorioretinopathy

**DOI:** 10.3390/ijerph17249455

**Published:** 2020-12-17

**Authors:** Yuh-Shin Chang, Shih-Feng Weng, Jhi-Joung Wang, Ren-Long Jan

**Affiliations:** 1 Department of Ophthalmology, Chi Mei Medical Center, Tainan 710, Taiwan; yuhshinchang@yahoo.com.tw; 2 Graduate Institute of Medical Sciences, College of Health Sciences, Chang Jung Christian University, Tainan 711, Taiwan; 3 Department of Healthcare Administration and Medical Informatics, Kaohsiung Medical University, Kaohsiung 807, Taiwan; also99@gmail.com; 4 Department of Medical Research, Kaohsiung Medical University Hospital, Kaohsiung 807, Taiwan; 5 Center for Medical informatics and Statistics, Office of R&D, Kaohsiung Medical University, Kaohsiung 807, Taiwan; 6 Department of Medical Research, Chi Mei Medical Center, Tainan 710, Taiwan; 400002@mail.chimei.org.tw; 7 Department of Anesthesiology, Chi Mei Medical Center, Tainan 710, Taiwan; 8 AI Biomed Center, Southern Taiwan University of Science and Technology, Tainan 710, Taiwan; 9 Department of Pediatrics, Chi Mei Medical Center, Liouying, Tainan 736, Taiwan

**Keywords:** central serous chorioretinopathy, longitudinal health insurance database, retrospective study, topical ophthalmic corticosteroid

## Abstract

This retrospective, nationwide, matched cohort study investigated the temporal relationship of central serous chorioretinopathy (CSCR) following topical ophthalmic corticosteroid (TOC) use. Using the Longitudinal Health Insurance Database 2000 (LHID2000), we collected patients diagnosed with CSCR between January 2001 and December 2010 (*n*  =  2921) and a control group (*n*  =  17,526). Information for each patient was collected and tracked from the index date until December 2011. TOC users were classified based on (i) the date of the last prescription before diagnosis: current users (≤30 days) and former users (31–182 days and ≥183 days) and (ii) the prescription refill intervals: persistent users (interval ≤90 days) and non-persistent users (interval >90 days). The odds ratio (OR) was estimated from multivariate conditional logistic regression after adjusting for relevant confounders. After adjusting for age, sex, geographic region, index date, previously known comorbidities, the date of last TOC prescription before diagnosis, or prescription refilling intervals, the results revealed that patients were likely to have developed CSCR while using TOCs currently (OR = 30.42, 95% CI = 25.95–35.66, *p <* 0.001) and persistently (OR = 7.30, 95% CI = 6.13–8.69, *p <* 0.001) as compared to the controls. Our results indicate that current or persistent TOCs use increases the risk of CSCR. Thus, patients requiring TOCs should be advised of this risk, particularly in current or persistent use conditions.

## 1. Introduction

Central serous chorioretinopathy (CSCR) is a major cause of vision threat characterized by serous neurosensory retinal detachments and/or retinal pigment epithelium (RPE) detachment [1,2]. These changes are mostly confined to the macula and are linked to the leakage of fluid from the RPE into the subretinal space. CSCR mostly affects adults, especially young and middle-aged men, who frequently exhibit a mild reduction in visual acuity over a short period of time. Other visual defects include relative central scotoma, micropsia, metamorphopsia, moderate dyschromatopsia, and reduced contrast sensitivity [3]. Clinically, acute CSCR can develop with spontaneous resolution within three to six months, recur episodically within the first year, and present chronically, with particular RPE changes, persevering shallow retinal detachment, and visual damage [4,5,6,7]. Although the pathophysiology and exact molecular mechanism of CSCR development remains poorly understood, advancement in imaging studies including optical coherence tomography, fluorescein angiography, indocyanine green angiography [8], and fundus autofluorescence [9] have highlighted the contribution of alterations in choroidal circulation and RPE function in CSCR pathogenesis.

Regardless of the lack of a clear understanding of the factors triggering CSCR, the condition is frequently related to the use of corticosteroids, including topical ophthalmic corticosteroid (TOC) usage [10,11,12]. Evidently, corticosteroid usage is related to CSCR through pathologic effects in the choriocapillaris or RPE. Corticosteroids may contribute to increased choriocapillaris permeability or RPE function impairment by enhancing platelet aggregation, leading to the formation of microthrombus, increased blood viscosity, and finally altered choroidal microcirculation [5]. Some reports suggesting an association between corticosteroids and microvasculature of the retina show that the effects of long-term corticosteroid usage include increased permeability and weakness of the choriocapillaris [13,14]. Recently, it has been proposed that corticosteroids contribute to choriocapillary hyperpermeability via a mechanism involving interaction with the mineralocorticoid receptor [15,16]. In addition, previous studies suggest that corticosteroids induce paraptosis in RPE cells, wherein cell death proceeds in a non-apoptotic, caspase-independent pathway [13,14].

CSCR has been reported to have an association with corticosteroid use through different modes of application including oral [11,17], intravenous [18], epidural [19,20], intra-articular [21], and even intranasal [22,23] or inhalation [24]. It is worth noting in particular that we have previously demonstrated the role of TOC administration in the development of CSCR [25]. To prevent further vision impairment, it is crucial to further assess the clinical history of TOC administration before the index date of CSCR diagnosis, based on the date of the last prescription or the time interval between prescription refills.

To our knowledge, this is the first report investigating the association between the current or persistent use of ophthalmic corticosteroids and CSCR, even though administration of TOCs is the primary direct route for ocular absorption. In this report, we used data from the Taiwan National Health Insurance Research Database (NHIRD) to appraise whether current or persistent TOC use increases the risk of CSCR.

## 2. Experimental Section

### 2.1. Database

The Taiwan National Health Insurance (NHI) program, launched on 1 March 1995, offers comprehensive medical care to nearly all (>99%) of the Taiwanese population. The National Health Insurance Research Database (NHIRD) collects encrypted identification numbers of patients including their sex, date of birth, dates of ambulatory visits, dates of hospital admission and discharge, details of pharmacological prescriptions, payments covered by NHI and their related International Classification of Diseases, Ninth Revision, Clinical Modification (ICD-9-CM) codes for physician’s diagnoses, examinations and procedures. One million beneficiaries with their claims data from 1996 to 2011 were randomly selected and systematically assigned to create a new subdata in 2000. The newly created Longitudinal Health Insurance Database 2000 (LHID2000) is a subdata of the NHIRD. The age, sex and average insured payroll-related premiums of this LHID2000 cohort were similar to all NHI enrollees.

### 2.2. Selection of Patients and Variables

Using the LHID2000, we collected data for patients (*n*  =  2921) diagnosed with CSCR (ICD-9-CM code 362.41), who either received ambulatory (including patients who made emergency department visits) or inpatient care between January 2001 to December 2010. The controls (*n*  =  17,526, the ratio of the number of CSCR patients to controls being 1 to 6) were patients without recorded CSCR diagnosis in this dataset. The ratio of one case to six controls is set to increase the statistical power as optimal as possible. Information for each patient in both groups was collected and tracked from the index date until December 2011. The controls were matched with the study of patients using propensity scores based on age, gender, geographic zone, and index date. The initial date of diagnosis of CSCR in a patient was defined as the index date, which was created for matched controls. For reducing selection bias, patients were excluded if they had been diagnosed with CSCR in ambulatory or inpatient care before January 2001. Furthermore, to erase possible etiologies of RPE leaks rather than CSCR, we excluded patients with previous diagnoses of the following diseases before enlistment: malignant neoplasm of the choroid (ICD-9-CM code 190.6), degenerative myopia (360.21), hemorrhagic RPE detachment (362.43), exudative age-related macular degeneration (362.52), macular hole (362.54), hereditary retinal dystrophies (362.7x), focal chorioretinitis (363.0x), disseminated chorioretinitis (363.1x), Harada’s disease (363.22), and angioid streak (363.43). The diagnoses of CSCR and other eye diseases were made by ophthalmologists after a series of eye examinations.

Prescription medications were identified using the National Drug Codes on the outpatient prescription drug claims. The TOCs administered included hydrocortisone, prednisolone, dexamethasone, betamethasone, and flumetholone. Patients using TOCs were deemed current users when the patients’ last prescription was dispensed within 30 days preceding the index date. The patients who had stopped receiving a prescription for TOCs between 31 and 182 days or greater than 182 days before the index date were considered former users. Persistent use was defined as the continuous and regular refilling of a TOC with a refilling interval less than or equal to 90 days based on the drug claim data. Non-persistent use by subjects was defined as an interval of greater than 90 days between TOC refills. The cut-off points for medication were determined based on clinical experience rather than on ROC curves, as has been proved valid in previous studies [17,26]. We divided the patients into six age groups based on their age-distribution pattern; this classification has also been proved valid in previous studies [17,25]. Furthermore, we classified the patients based on gender and geographic region to evaluate the influence of gender or region on CSCR.

Based on reported risk factors for CSCR, the following medical comorbidities and their ICD-9-CM codes were included in this study: diabetes mellitus (250), hyperlipidemia (272), psychiatric disease (292–311 except 303), hypertension (401–405), coronary artery disease (410–414), allergic rhinitis (477), asthma (493), chronic renal disease (582, 583, 585, 586, 588), and peptic ulcer (531–534) [11,17,27,28,29,30]. A search for these comorbidity diagnostic codes was made in the LHID2000. Patients registering with one of the above ICD-9-CM codes within 12 months prior to the index date were identified and further verified by a record of at least three ambulatory visits.

The institutional review board at the Taiwan Chi Mei Medical Center ruled that this research complied with the tenets of the Declaration of Helsinki and was eligible for an exemption from review. Since the analyzed datasets were obtained from a database devoid of personally identifiable information the requirement for informed consent was waived.

### 2.3. Statistical Analysis

The SAS statistical software, version 9.3.4 of the SAS System for Windows (SAS Institute Inc., Cary, NC, USA) was used for all statistical analysis. Baseline characteristics of these patients were compared using chi-square tests, and Student’s *t*-test was employed for continuous variables. The Cochran–Armitage trend test was used for ordinal variables. Furthermore, using chi-square tests, we compared current versus former and persistent versus non-persistent users of TOCs from CSCR patients and control groups. Univariate conditional logistic regression (conditional on index date, age, gender, and geographic zone) was used to estimate the odds ratio (OR) for TOC use for various “most recent” prescription times before the index date and refilling intervals between CSCR patients and controls. The adjusted OR was estimated from multivariate conditional logistic regression after adjusting for relevant comorbidities. The data were also adjusted for differences in the date of the last prescription before the index date or refilling intervals of the corticosteroid. The significant-difference threshold level was set at *p <*  0.05.

## 3. Results

### 3.1. Demographic Data

In total, data from 2921 patients with CSCR and 17,526 controls were collected after excluding ineligible patients. Table 1 shows that the average ages of the CSCR patients and the controls were 42.26 ± 14.57 and 42.34 ± 14.35 years, respectively. These patients were divided into six groups based on their age. Of the 2921 CSCR patients, 289 (9.89%) were < 25 years, 705 (24.14%) were between 25–34 years, 822 (28.14%) were between 35–44 years, 542 (18.56%) were between 45–54 years, 320 (10.96%) were between 55–64 years, and 243 (8.32%) were ≥ 65 years of age. In the CSCR group, 1248 (42.73%) patients were women and 1673 (57.27%) were men. The geographic distribution of the CSCR patients was as follows; northern (1024; 35.06%), central (1218; 41.70%), southern (613; 20.99%), and eastern region (66; 2.26%).

### 3.2. Associated Risk Factors

Table 2 shows the adjusted ORs of current or past TOC use between CSCR patients and controls after conditioning the data on age-group, sex, geographic region, and the year of the index date. Data were also adjusted for relevant comorbidities and the differences in the date of the last prescription before the index date. The adjusted OR for patients with CSCR who used TOCs ≤ 30 days before the index date (i.e., “current users”) was 30.42 (95% CI  =  25.95–35.66, *p <*  0.001) compared to controls, after adjusting for other confounding factors. In addition, the adjusted ORs for CSCR patients who had used TOCs 31–182 days before the index date (i.e., “former users”) was 2.33 (95% CI  =  1.99–2.73, *p <* 0 .001), and the adjusted OR for CSCR patients who had used TOCs ≥ 183 days before the index date (i.e., also “former users”) was 2.03 (95% CI  =  1.71–2.41, *p <* 0 .001). After stratifying CSCR and control groups according to the comorbidities, we found that the adjusted ORs in CSCR with peptic ulcer or coronary artery disease were statistically significant (adjusted OR = 1.48, 95% CI = 1.19–1.84 for peptic ulcer and adjusted OR = 1.36, 95% CI = 1.023–1.809 for coronary artery disease).

Table 3 reports the adjusted ORs of TOC use for various refilling intervals for CSCR patients and controls after conditioning the data on age-group, gender, geographic zone, and the year of index date, adjusted for relevant comorbidities, and the different refilling intervals of corticosteroids use. The adjusted OR for patients with CSCR, who were persistent TOC users (a refilling interval ≤ 90 days), was 7.30 (95% CI  =  6.13–8.69, *p <* 0 .001) compared to controls after adjustment for other confounding factors. Besides, 279 out of 2921 CSCR patients (9.55%) were non-persistent TOC users (a refilling interval > 90 days, adjusted OR  =  5.80, 95% CI  =  5.27–6.39, *p*  = 0 .004). They are less than persistent users (*n* = 1094, 37.45%) but they remained at significant risk compared to non-drug users after conditional logistic regression analysis (Table 3). After stratifying CSCR and control groups according to the comorbidities, we found that the adjusted ORs in CSCR with peptic ulcer or coronary artery disease were statistically significant (adjusted OR = 1.36, 95% CI = 1.11–1.66 for peptic ulcer and adjusted OR = 1.33, 95% CI = 1.02–1.72 for coronary artery disease).

## 4. Discussion

To the best of our knowledge, our research is the first large-scale retrospective, case-controlled study that evaluates the association between current or persistent TOC use and CSCR. Our results show that patients who are current or persistent users of TOCs were at a higher risk of developing CSCR compared to the general population.

Changes in the choroidal circulation and dysregulation of RPE function are involved in the pathogenesis of CSCR, although the development of CSCR in totality is poorly understood. The exact role of corticosteroids in the pathogenesis of CSCR is unclear; however, corticosteroids have been hypothesized to play an important role. Proposed mechanisms by which corticosteroids alter choroidal microcirculation include their tendency to augment platelet aggregation and subsequent formation of microthrombus and elevated blood viscosity [5]. Recent reports indicating an association between corticosteroids and the retinal microvasculature show that the long-term effects of corticosteroid use include hyperpermeability and fragile choriocapillaris [13,18,31]. Additionally, a mechanism was proposed suggesting that corticosteroids might contribute to a hyperpermeable choriocapillary via the mineralocorticoid receptor [15,16]. Corticosteroids, by altering ion and water transport and inhibiting collagen formation, may lead to ion pumping dysfunction in the epithelium [31]. Furthermore, Torriglia et al. showed that corticosteroids induce a decline in mitochondrial activity and elicit paraptosis in RPE cells that compose the outer blood–retinal barrier, thus leading to subretinal fluid accumulation [13,14].

In our previous study, we observed a relationship between CSCR and the various modes of corticosteroid use, including topical, oral, nasal spray and injection routes [25]. The topical administration of ophthalmic corticosteroids is the most direct route for delivery to the ocular tissue and they may affect the posterior segment either through the transconjunctival or the transcorneal pathways [32,33]. While prescribing them, ophthalmologists should exercise extra caution and inform patients of the risk of CSCR.

In this study, we further investigated the association between the CSCR and topical corticosteroids use; current (the date of the last prescription before diagnosis ≤ 30 days) or persistent (a refilling interval ≤ 90 days). TOCs are some of the most potent and effective modalities to control inflammation in different ocular conditions, such as ocular surface disease, immune-mediated corneal diseases, anterior uveitis, and postoperatively [34]. Considering the therapeutic concentrations, the TOCs route is inefficient to deliver pharmaceuticals to the posterior segment of the eye, because of anatomical and physiological constraints of the eye, such as rapid drainage through the nasolacrimal ducts, low permeability of the corneal epithelium, systemic absorption, and the blood–aqueous barrier [35]. However, with respect to the drug distribution, the topical route can be used to deliver corticosteroids to the posterior eye through corneal, conjunctival, scleral, uveal, and choroidal pathways [32,33,36,37,38]. We hypothesize that the use of TOCs currently or persistently will result in higher corticosteroid concentration in the posterior pole and thus will be associated with greater risk of CSCR development.

The most important strength of our study was to expand the primary study criteria of TOC usage to include the date of the last prescription before diagnosis and refilling intervals of corticosteroid usage, as identified through National Drug Codes on outpatient prescription drug claims. Additionally, our study was based on a large nationwide population-based dataset ensuring that the selection bias due to different clinical centers was not an issue of debate. The NHIRD comprises electronically registered claims data, thus obviating recall bias and ensuring accuracy. Previous studies using the same database have proved its validity [39,40]. Furthermore, our study has merged 10 years of longitudinal data on recently administered TOCs, including current or persistent use of corticosteroids in a cohort with CSCR patients and controls. Our results, assessing the association between TOCs and CSCR are reliable because the ORs were determined with appropriate adjustments for potentially confounding variables.

At the same time, our study has several limitations. For instance, the medical history of the sample groups before January 1996 was not available. Therefore, the incidence of CSCR in the control group before January 1996 could not be verified, potentially compromising findings. Additionally, despite the fact that the ICD-9 code 362.41 is rather specific and typical CSCR is relatively easy to diagnose, analysis of the imaging studies was not possible using the LHID2000, and thus the diagnoses could not be verified. In order to alleviate this difficulty, this study excluded cases of RPE leaks resulting from causes other than CSCR to mitigate the possible effect of misdiagnosis. Notably, similar analyses in previous reports have been used to identify CSCR and have proved to be valid while using similar exclusion criteria [25,41]. Information regarding personal characteristics such as psychological stress, which might contribute to CSCR, were not available in the LHID2000, which possibly compromises our results. Thus, we included psychiatric disease as a confounding factor to mitigate this problem. Finally, we have not classified the TOCs into different formulations. We cannot, therefore, comment on which of the formulations has the highest association with CSCR.

## 5. Conclusions

In summary, patients with a history of TOC use within one year before the index date of CSCR diagnosis, especially current or persistent users, were at a higher risk of developing CSCR than controls, after adjusting for relevant comorbidities. Importantly, ophthalmologists should be cautious while prescribing TOCs—especially under current or persistent use conditions—and inform the patients of the risk of CSCR.

## Figures and Tables

**Table 1:** Baseline characteristics of central serous chorioretinopathy (CSCR) patients and control group patients after propensity score matching.

	CSCR *n* = 2921	Controls *n* = 17,526	*p* Value
	Number (%)	Number (%)	
**Age (years; Mean ± SD)**	42.26 ± 14.57	42.34 ± 14.35	0.7905 ^a^
**Age (years)**			
<25	289 (9.89)	1663 (9.49)	0.6533 ^b^
25–34	705 (24.14)	4168 (23.78)	
35–44	822 (28.14)	4976 (28.39)	
45–54	542(18.56)	3383 (19.30)	
55–64	320 (10.96)	1951 (11.13)	
≥65	243 (8.32)	1385 (7.90)	
**Gender**			
Male	1673 (57.27)	10081 (57.52)	0.8039 ^c^
Female	1248 (42.73)	7445 (42.48)	
**Geographic region**			
Northern	1024 (35.06)	6144 (35.06)	0.8789 ^c^
Central	1218 (41.70)	7264 (41.45)	
Southern	613 (20.99)	3678 (20.99)	
Eastern	66 (2.26)	440 (2.51)	

^a^ Student’s *t*-test; ^b^ Cochran-Armitage trend test; ^c^ Chi-square test. Abbreviations: CSCR: central serous chorioretinopathy; SD: standard deviation.

**Table 2:** Odds ratios (OR) were obtained by univariate and multivariate logistic regression for various prescription times of topical ophthalmic corticosteroids use between CSCR patients and controls.

Topical Ophthalmic Corticosteroids	CSCR *n* = 2921	Controls *n* = 17,526	Crude OR (95% CI)	*p* Value	Adjusted OR ^a^ (95% CI)	*p* Value
**The last prescription time before index date (days)**	Number (%)	Number (%)				
≤30	934 (31.98)	296 (1.69)	31.39 (26.81–36.75)	<0.001	30.42 (25.95–35.66)	<0.001
31–182	247 (8.46)	1013 (5.78)	2.50 (2.14–2.91)	<0.001	2.33 (1.99–2.73)	<0.001
≥183	192 (6.57)	918 (5.24)	2.13 (1.79–2.52)	<0.001	2.03 (1.71–2.41)	<0.001
None	1548 (53.00)	15,299 (87.29)	1.00		1.00	
**Comorbidity**						
Hypertension	347 (11.88)	1582 (9.03)	1.45 (1.27–1.66)	<0.001	1.10 (0.92–1.32)	0.2796
Diabetes	175 (5.99)	770 (4.39)	1.43 (1.20–1.70)	<0.001	1.01 (0.80–1.27)	0.9158
Hyperlipidemia	153 (5.24)	641 (3.66)	1.48 (1.23–1.79)	<0.001	1.21 (0.96–1.54)	0.1151
Chronic renal disease	39 (1.34)	127 (0.72)	1.88 (1.30–2.71)	<0.001	1.53 (0.98–2.40)	0.0606
Peptic ulcer	162 (5.55)	584 (3.33)	1.73 (1.44–2.07)	<0.001	1.48 (1.19–1.84)	0.0005
Psychiatric disease	174 (5.96)	756 (4.31)	1.42 (1.19–1.68)	<0.001	1.21 (0.99–1.48)	0.0822
Allergic respiratory disease	143 (4.90)	509 (2.90)	1.73 (1.43–2.09)	<0.001	1.11 (0.86–1.42)	0.4393
Coronary artery disease	106 (3.63)	405 (2.31)	1.65 (1.31–2.07)	<0.001	1.36 (1.023–1.809)	0.0343

^a^ Adjusted OR was counted by conditional logistic regression that was conditioned on sex, age-group, geographic region, and the year of index date, and then OR was adjusted by diabetes, hypertension, hyperlipidemia, peptic ulcer, chronic renal disease, psychiatric disease, allergic respiratory disease, coronary artery disease, and the last prescription time before index date of topical ophthalmic corticosteroids. Abbreviations: CSCR: central serous chorioretinopathy; CI: confidence interval; OR: odds ratio.

**Table 3:** OR obtained by univariate and multivariate logistic regression for various prescription durations of topical ophthalmic corticosteroids use between CSCR patients and controls.

Topical Ophthalmic Corticosteroids	CSCR *n* = 2921	Controls *n* = 17,526	Crude OR (95% CI)	*p* Value	Adjusted OR ^a^ (95% CI)	*p* Value
**Refilling intervals (days)**	Number (%)	Number (%)				
Interval ≤ 90	1094 (37.45)	1853 (10.57)	7.95 (6.70–9.44)	<0.001	7.30 (6.13–8.69)	<0.001
Interval > 90	279 (9.55)	374 (2.13)	6.02 (5.48–6.62)	<0.001	5.80 (5.27–6.39)	0.0044
None	1548 (53.00)	15,299 (87.2)	1.00		1.00	
**Comorbidity**						
Hypertension	347 (11.88)	1582 (9.03)	1.45 (1.27–1.66)	<0.001	1.07 (0.91–1.26)	0.4007
Diabetes	175 (5.99)	770 (4.39)	1.43 (1.20–1.70)	<0.001	1.11 (0.90–1.36)	0.3378
Hyperlipidemia	153 (5.24)	641 (3.66)	1.48 (1.23–1.79)	<0.001	1.10 (0.89–1.37)	0.3727
Chronic renal disease	39 (1.34)	127 (0.72)	1.88 (1.30–2.71)	<0.001	1.41 (0.94–2.11)	0.0994
Peptic ulcer	162 (5.55)	584 (3.33)	1.73 (1.44–2.07)	<0.001	1.36 (1.11–1.66)	0.0033
Psychiatric disease	174 (5.96)	756 (4.31)	1.42 (1.19–1.68)	<0.001	1.12 (0.93–1.36)	0.2277
Allergic respiratory disease	143 (4.90)	509 (2.90)	1.73 (1.43–2.09)	<0.001	1.19 (0.95–1.49)	0.1354
Coronary artery disease	106 (3.63)	405 (2.31)	1.65 (1.31–2.07)	<0.001	1.33 (1.02–1.72)	0.0352

^a^ Adjusted OR was counted by conditional logistic regression that was conditioned on sex, age-group, geographic region, and the year of index date, and then OR was adjusted by diabetes, hypertension, hyperlipidemia, peptic ulcer, chronic renal disease, psychiatric disease, allergic respiratory disease, coronary artery disease, and refilling intervals of topical ophthalmic corticosteroids. Abbreviations: CSCR: central serous chorioretinopathy; CI: confidence interval; OR: odds ratio.
